# Evaluating the Correlation between Serum NT-proBNP Level and Diastolic Dysfunction Severity in Beta-Thalassemia Major Patients

**Published:** 2016-04-13

**Authors:** Behzad Alizadeh, Zahra Badiee, Mahmoud Mahmoudi, Mahsa Mohajery

**Affiliations:** 1 *Emam Reza Hospital, Mashhad University of Medical Sciences, Mashhad, Iran.*; 2 *Doctor Sheikh Hospital, Mashhad University of Medical Sciences, Mashhad, Iran.*

**Keywords:** *Beta-thalassemia*, *Pro-brain natriuretic peptide*, *Ventricular dysfunction, left*

## Abstract

**Background:** N-terminal pro-brain natriuretic peptide (NT-proBNP) is a sensitive biomarker for the detection of asymptomatic left ventricular (LV) dysfunction. Since β-thalassemia major patients suffer from early diastolic dysfunction due to iron deposition of chronic blood transfusion, we tried to evaluate the correlation between the serum NT-proBNP level and the severity of LV diastolic dysfunction determined by echocardiography in these patients.

**Methods:** Fifty β-thalassemia major patients with normal LV systolic function were studied by tissue Doppler echocardiography, and blood samples were taken at the same time to measure the serum NT-proBNP level. Using flow velocity through the mitral valve on the tissue velocity of the mitral annulus in early ventricular filling (E/E') as an LV diastolic function indicator, the patients were divided into 3 groups: group 1) no diastolic dysfunction (E/E' < 8), group 2) suspected diastolic dysfunction (E/E' = 8-15), and group 3) documented diastolic dysfunction (E/E' >15). Other variables assessed included sex, age, method of chelator therapy, and mean hemoglobin and ferritin levels for the past 2 years.

**Results:** According to the echocardiographic findings of all the 50 patients (29 male and 21 female) with an age range of 11-35 years (mean = 17.98 y), 46% were classified in group 1, 54% in group 2, and none in group 3. The NT-proBNP level was 1070 ± 566 ng/mL in group 1 and 974 ± 515 ng/mL in group 2. The t-test showed no significant difference between groups 1 and 2 in the NT-proBNP level (p value = 0.536).

**Conclusion:** Due to specific conditions in thalassemia major patients, the correlation between the serum NT-proBNP level and the severity of diastolic dysfunction seems to be not meaningful.

## Introduction

Beta thalassemia is one of the most important inherited hematologic disorders and is developed due to a reduction in or absence of β-globin chain synthesis.^1^ Patients with β-thalassemia major present with severe hemolytic anemia during the first 2 years of life and need lifelong blood transfusion in order to survive.^2^^, ^^3^ The hemolysis of red blood cells and repeated blood transfusion cause iron overload in different organs, especially the heart.^4^^, ^^5^ Despite recent achievements in chelation therapy, heart failure resulted from iron deposition in the myocardium is still the main cause of death in β-thalassemia major patients.^6^ Left ventricular (LV) diastolic heart failure precedes systolic failure, and it occurs early in life in these patients.^7^

The inability of the LV to obtain blood in low filling pressures is the main problem in diastolic dysfunction.^8^ Doppler echocardiography is the method of choice in the diagnosis of diastolic dysfunction in routine clinical settings.^9^

Brain natriuretic peptide (BNP) is a biomarker secreted from stretched LV myocytes in response to increased cardiac volume and pressure.^10^ The expression of the BNP gene, which is located on chromosome 1, produces proBNP, a 108-amino-acid peptide. This peptide is hydrolyzed to active 32-amino-acid BNP and inactive 76-amino-acid NT-proBNP by the furin enzyme at the time of secretion into blood.^11^ Both BNP and NT-proBNP are sensitive biomarkers in finding asymptomatic LV dysfunction.^12^ However, their sensitivity is low in diagnosing diastolic dysfunction in comparison with systolic dysfunction. It has been demonstrated that, by comparison to BNP, NT-proBNP has a high predictive value and low interaction with other markers in blood.^13^ Moreover, its half-life is higher and its blood level fluctuation rate is slower than those of BNP.^14^^, ^^15^

In the present study we sought to measure the serum NT-proBNP level in β-thalassemia major patients as a disease with early isolated diastolic dysfunction and evaluate the correlation between the NT-proBNP level and the severity of LV diastolic dysfunction determined by echocardiography.

## Methods

This cross-sectional study was conducted from July to December 2013. The study protocol was approved by the Ethics Committee of Mashhad University of Medical Sciences. After reviewing the medical files of more than 200 β-thalassemia major patients who were visiting our outpatient thalassemia clinic regularly, we selected 50 patients via purposive sampling. These patients all met our inclusion criteria, including preserved LV ejection fraction (> 50%), and none of them had any of the exclusion criteria, comprising systolic heart failure, more-than-moderate valvular heart disease, rhythm disturbances, acute or chronic pulmonary disease, and impaired thyroid, renal, or liver function. Informed consent was obtained from all the patients before enrollment in the study. 

All the patients underwent a thorough echocardiographic study by a pediatric cardiologist, with a Vivid 3 echocardiographic machine equipped with a standard Probe 3s (General Electric Company [GE], NY, USA), between 5 and 10 days after blood transfusion. Standard 2-dimensional views as well as pulse and tissue Doppler were obtained in the parasternal and lateral decubitus positions from the mitral valve tip and annulus.

LV diastolic function was evaluated by measuring the velocity of the blood flow through the mitral valve, and the tissue velocity of the mitral annulus was determined at the beginning of diastole (velocity of the blood flow through the mitral valve at the beginning of diastole [E] and tissue velocity of the mitral annulus at the beginning of diastole [E´]) and after atrial contraction (velocity of the blood flow through the mitral valve after atrial contraction [A] and tissue velocity of the mitral annulus after atrial contraction [A´]). Blood velocity through the mitral valve reflects ventricle motion in the longitudinal axis and constitutes an important parameter in the systolic and diastolic function of the LV.^16^

The patients were divided into 3 groups based on the velocity of the mitral inflow E wave to E′ mitral annular velocity ratio (E/E´). Since in high filling pressures, E´ remains low but E shows an increase, E/E´ in the mitral valve is related to LV filling pressure and pulmonary capillary wedge pressure (PCWP). When E/E´ is greater than 15, PCWP tends to exceed 20 mmHg and when E/E´ is less than 8, PCWP tends to be normal.^17^ The E/E´ ratio has been used in different studies for the evaluation of LV diastolic dysfunction; accordingly, we decided to employ this ratio to categorize our patients into 3 groups: group 1) no diastolic dysfunction (E/E' < 8), group 2) suspected diastolic dysfunction (E/E' = 8-15), and group 3) documented diastolic dysfunction (E/E' > 15).

Blood samples were drawn from all the patients at the time of echocardiography. After the samples were centrifuged at 4000 c/min for 10 minutes, the serum was extracted and stored at -20 °C. When all the samples were obtained, the serum NT-proBNP level was determined using the Human N-terminal pro-Brain Natriuretic Peptide ELISA Kit (Bioassay Technology Laboratory, China). Other variables, including sex, age, method of chelator therapy, mean hemoglobin level for the past 2 years, and mean ferritin level for the past 2 years were extracted from the patients’ documents in the clinic.

The data were analyzed using SPSS, version 10.0 (IBM, Armonk, USA). The t-test was conducted for the comparison of the quantitative variables and the chi-square test for the qualitative variables. The correlation between the variables was determined using the Pearson coefficient. A p value < 0.05 was considered statistically significant.

## Results

A total of 50 (29 male and 21 female) β-thalassemia major patients at a mean age of 17.98 years (11–35 y) were included in this study. The patients were divided into 3 groups based on the mitral inflow E wave velocity to E′ mitral annular velocity ratio (E/E´): group 1) no diastolic dysfunction (E/E´< 8), group 2) suspected diastolic dysfunction (E/E´ = 8-15), and group 3) documented diastolic dysfunction (E/E´> 15). Twenty-seven (46%) patients were categorized in group 1 and 23 (54%) in group 2. There was no patient in group 3 in our study. The baseline demographic information and measured echocardiographic variables are presented in Table 1. 

The chi-square test showed no correlation between sex and diastolic dysfunction (p value > 0.05). The mean ± SD of age was 17.59 ± 3.905 in group 1 and 18.43 ± 5.273 in group 2. Although the mean age was higher in group 2 (as was expected), there was no statistically meaningful difference in age between the 2 groups. The mean level of NT-proBNP in serum was 1070.52 ng/L in group 1 and 974.30 ng/L in group 2. The t-test showed no significant difference in the NT-proBNP level between the 2 groups (p value > 0.05).

All our patients were under chelation therapy. Eighteen patients used Desferal^®^, 9 Osveral^®^, and 23 Desferal^®^ + L1. Additionally, 44% of the patients using Desferal^®^ and Osveral^®^ had suspected diastolic dysfunction, and 48% of those using Desferal^®^ + L1 were in the suspected group. These data demonstrated that there was no correlation between the chelation therapy method and the onset of diastolic dysfunction (p value > 0.05). Moreover, the one-way ANOVA test showed no significant difference in the NT-proBNP level between the groups of patients treated with different chelation therapy methods (p value > 0.05). There was no significant correlation between the NT-proBNP level and sex based on the t-test (p value > 0.05) as well as between the NT-proBNP level and age based on the Pearson coefficient (r = 0.206, p value = 0.152).

The mean hemoglobin level before transfusion over the past 2 years was 9.74 ± 0.74 gr/dL in the group with no diastolic dysfunction and 9.42 ± 0.67 gr/dL in the group with suspected diastolic dysfunction. The Pearson coefficient showed a significant correlation between the mean hemoglobin level before transfusion in the last 2 years and the NT-proBNP level (r = 0.309, p value = 0.029). On the other hand, although the mean hemoglobin level was higher in group 1, the t-test showed no statistically significant difference in the mean hemoglobin level between the 2 groups (p value > 0.05).

 The mean serum ferritin level in the past 2 years was 4835.71 ± 2242.54 ng/mL in the group without diastolic dysfunction and 5679.39 ± 3612.34 ng/mL in the group with suspected diastolic dysfunction, and the difference between the 2 groups was not significant statistically (p value > 0.05). Moreover, no correlation was detected between the mean serum ferritin level and the NT-proBNP level between the 2 groups (p value > 0.05) (Table 1).

**Table 1:** Patients’ characteristics

**Variable**	**Suspected Diastolic Dysfunction**	**No Diastolic Dysfunction**	**P value**
**Male/Female**	**8/15**	**13/14**	** 0.350**
**Age (y)**	**18.43 ± 5.27**	**17.59 ± 3.90**	** 0.520**
**Hemoglobin ** **(g/dL)**	**9.42 ± 0.67**	**9.74 ± 0.74**	** 0.118**
**Ferritin (ng/ml)**	**56.79 ± 36.12**	**48.35 ± 22.42**	** 0.319**
**Ejection fraction (%) **	**60.60 ± 6.35**	**61.44 ± 6.12**	** 0.638**
**Left atrial diameter (mm)**	**32.02 ± 6.09**	**31.98 ± 5.62**	** 0.984**
**End-diastolic left ventricular diameter (mm)**	**45.66 ± 6.65**	**46.20 ± 5.31**	** 0.752**
**End-systolic left ventricular diameter (mm)**	**29.64 ± 4.62**	**29.79 ± 4.47**	** 0.911**
**End-diastolic left ventricular volume (cm** ^3^ **)**	**79.54 ± 21.57**	**84.52 ± 20.61**	** 0.408**
**End-systolic left ventricular volume (cm** ^3^ **) **	**32.93 ± 12.32**	**34.64 ± 8.96**	** 0.573**
**TAPSE**	**2.19 ± 0.36**	**2.28 ± 0.27**	** 0.331**
**E** **(cm/s)**	**116.21 ± 14.66**	**99.70 ± 14.05**	**< 0.001**
**E´ (cm/s)**	**12.52 ± 1.87**	**14.37 ± 1.84**	**< 0.001**
**E/E´**	**9.41 ± 1.50**	**6.96 ± 0.76**	**< 0.001**
**A** **(cm/s)**	**63.17 ± 15.63**	**58.62 ± 12.71**	** 0.263**
**A´ (cm/s)**	**6.21 ± 1.80**	**6.70 ± 1.85**	** 0.355**
**E/A**	**1.94 ± 0.51**	**1.75 ± 0.38**	** 0.162**
**E´/A´**	**2.23 ± 0.69**	**2.31 ± 1.79**	** 0.693**

TAPSE, Tricuspid annular plane systolic excursion; E, Velocity of the blood flow through the mitral valve at the beginning of diastole E´, Tissue velocity of the mitral annulus at the beginning of diastole; A, Velocity of the blood flow through the mitral valve after atrial contraction; A´, Tissue velocity of the mitral annulus after atrial contraction

## Discussion

This study was the first study to evaluate the correlation between the NT-proBNP level and the Doppler echocardiographic diastolic indices of the LV in Iran and also the first one to assess the plasma level of this biomarker in thalassemia patients in Iran. Moreover, the use of BNP and NT-proBNP as diagnostic markers of systolic and diastolic heart failure is a new method, and studies evaluating them in thalassemia patients are very few worldwide. 

The mean age for our study population was 17.98 years, which shows improvement in the treatment plans of thalassemia patients insofar as none of them had systolic dysfunction yet. The mean age was 17.59 ± 3.90 in group 1 and 18.43 ± 5.27 in group 2, which was predictable since the probability of heart failure incidence increases as patients grow older. However, the difference as regards age between the 2 groups was not statistically significant. 

Echocardiography is the gold standard for evaluating diastolic function; nonetheless, it is not free from errors. Mitral flow velocity changes with any alteration in heartbeat, preload and afterload, heart’s contractility, and valve regurgitation.^18^ Moreover, in the clinical setting, detailed echocardiographic data are not applicable. Furthermore, echocardiographic data are somewhat operator-dependent and clinical trials need an objective and standard parameter for evaluation.^19^ A simple and rapid blood test which shows diastolic dysfunction, therefore, has a high clinical value.

Our results did not show a statistically meaningful difference in the NT-proBNP level between the patients with E/E' < 8 and those with E/E' = 8–15 (groups 1 and 2). Some other studies have reported similar results as well. In a study by Grewal et al.,^19^ since there was no significant difference in the NT-proBNP level between the patients without diastolic dysfunction and those with mild diastolic dysfunction, all these patients were categorized in 1 group. Based on a study by Aessopos et al.^20^ in Greece on thalassemia major patients, BNP was significantly higher in the patients with severe heart failure (New York Heart Association [NYHA] class 3 or 4), while there was no such difference in the patients in NYHA class 2 and those with normal systolic function. That study showed that even after the development of systolic dysfunction, there was not much difference in the BNP level in the thalassemia patients until the advanced clinical stages and suggested that the potential deficiency of BNP-related neurohormonal mechanisms might be responsible for impairing the clinical usefulness of this biomarker in β-thalassemia major patients. In a study by Kremastinos et al.,^21^ the difference in the NT-proBNP level between the patients without diastolic dysfunction (E/E’ < 8) and those with suspected diastolic dysfunction (E/E’ = 8-15) was statistically meaningful; however, the numerical difference was not considerable and, compared to the significant increase in the NT-proBNP level in the patients with documented diastolic dysfunction (E/E' > 15), was not significant.

It has been demonstrated that in asymptomatic patients with normal-to-mild diastolic dysfunction on echocardiography, LV pressure rises during activity.^22^ This can explain the rise in the NT-proBNP level in our thalassemia patients without diastolic dysfunction on echocardiography. Additionally, since NT-proBNP has a long half-life, its rise because of daily activities can be detected before E/E´ alters in resting condition.

The reported value for NT-proBNP in healthy people varies in different studies. Grewal et al.^19^ stated that the NT-proBNP level was < 70 ng/L in 90% of their healthy subjects, whereas McDonagh et al.^23^ reported that the highest level of NT-proBNP in a healthy person was approximately 123 ng/L. The NT-proBNP level in our patients, even in the group without diastolic dysfunction, was highly elevated; this elevation must be due to the specific conditions of thalassemia patients, including their chronic anemia.

Our findings illustrated a significant correlation between the mean hemoglobin level before transfusion (9.59 ± 0.72 gr/dL) in the last 2 years and the NT-proBNP level (1026 ± 540 ng/L) (r = 0.309, p value = 0.029). Studies which have evaluated the correlation between anemia and the NT-proBNP level have shown that NT-proBNP is elevated in anemic patients. Willis et al.^24^ demonstrated that NT-proBNP rose in their anemic patients without heart failure independent of gender and LV hypertrophy. Hogenhuis et al.^25^ showed that anemia elevated the NT-proBNP level independently of the severity of heart failure.

Cardiac iron overload can influence the secretory function of myocytes; it can, therefore, disturb the secretion of NT-proBNP at multiple points and result in inappropriate NT-proBNP levels in the serum.^20^ A wide range of factors can affect myocardial iron deposition and, thus, NT-proBNP levels. Some of these factors include the blood transfusion method, chelation therapy method, efficacy of the administered iron chelators, and patients’ compliance with their medications. Differences detected in NT-proBNP levels in different populations of β-thalassemia major patients can be due to such factors that usually vary based on the country and the culture where the patient lives.

The major limitation in the present study is its small sample volume. Nevertheless, it should be borne in mind that the thalassemia population is grown older in Iran, rendering finding this group of patients with preserved LV ejection fraction ever more challenging. 

## Conclusion

Due to specific conditions in β-thalassemia major patients, including chronic anemia, which results in the elevation of NT-proBNP independently, and iron deposition, which interferes with secretory pathways in cardiac cells, we could not find any correlation between the serum NT-proBNP level and diastolic dysfunction severity. As a result, it seems that NT-proBNP is not a useful marker in β-thalassemia major patients for the determination of early LV diastolic dysfunction.

## Notes:

***This paper should be cited as:*** *Alizadeh B, Badiee Z, Mahmoudi M, Mohajery M. Evaluating the Correlation between Serum NT-proBNP Level and Diastolic Dysfunction Severity in Beta-Thalassemia Major Patients. J Teh Univ Heart Ctr 2016;11(2):68-72.*
